# Promotion of BZW2 by LINC00174 through miR-4500 inhibition enhances proliferation and apoptosis evasion in laryngeal papilloma

**DOI:** 10.1186/s12935-020-01559-3

**Published:** 2020-09-29

**Authors:** Jiajia Liu, Tao Yang, Ying Zhang, Shuhui Wang

**Affiliations:** Department of Otolaryngology-Head and Neck Surgery, The Second Xiangya Hospital, Central South University, Changsha, 410011 Hunan China

**Keywords:** Laryngeal papillomatosis, BZW2, miR-4500, LINC00174, Apoptosis

## Abstract

**Background:**

We aimed to explore the roles of basic leucine zipper and W2 domains (BZW) 2 in the human papillomavirus-infected laryngeal papillomatosis.

**Methods:**

In the present study, BZW 2 knockdown and overexpressed cell lines were constructed. CCK-8 and colony formation assays were used to determine cell proliferation. Caspase-3 activity and nucleosomes fragmentation assays were used to determine cell apoptosis. qRT-PCR and Western blot were employed to evaluate the mRNA and protein levels of target genes, respectively. Luciferase and biotin-coupled miRNA pulldown assays were used to examine the interactions between mRNA and mRNA.

**Results:**

We observed the levels of BZW2 were up-regulated in the laryngeal papilloma (LP) tissues as compared with adjacent tissues. The knockdown of BZW2 significantly inhibited cell proliferation and promoted cell apoptosis in the LP cells. Additionally, we identified the expressions of BZW2 negatively regulated by miR-4500. Luciferase and biotin-coupled miRNA pulldown assays demonstrated that LINC00174 competed with the BZW2 for binding with miR-4500. Moreover, the results showed that LINC00174/miR-4500/BZW2 axis regulated cell proliferation and apoptosis.

**Conclusion:**

Our results demonstrated that the regulation of LINC00174/miR-4500/BZW2 axis might be used as an effective strategy for treatment of human papillomavirus-infected laryngeal papillomatosis.

## Background

Laryngeal papillomatosis (LP) is a rare disease caused by human papillomavirus (HPV) infection. As a benign squamous epithelial tumor occurred in the larynx, the LP is divided into juvenile and adult types based on age [1, 2]. Although its incidence is low, it is one of the major risk factors for children hoarseness. Additionally, the occurrence of LP also induces short of breath, chronic cough, and even asthma. When the LP spreads, it is difficult to remove all tumor tissues, thereby leading to its recurrence [2]. Besides, the recurrence of LP leads to its resistance to cell apoptosis [3]. Therefore, it is of great importance to understand the mechanisms underlying the occurrence and development of LP.

MicroRNAs (miRNAs) have been implicated to play an important role in various biological processes including cell migration, proliferation, invasion and apoptosis [4–6]. Since the discovery of miRNAs, a growing body of evidences support that they are associated with a series of diseases including cardiovascular disease, neurological disease, and many types of cancers [6–9]. The expressions of miRNAs are regulated by other miRNAs or transcription factors when the cells encounter stimuli [7]. Previous studies have demonstrated that miRNAs serve as important biomarkers in LP. It is worthwhile to identify miRNAs with regulatory effects in the occurrence and development of LP.

Basic leucine zipper and W2 domains (BZW) 2 are encoded by *BZW2*. It is known that BZW regulates the transcriptions of histone H4 [10]. Additionally, high expressions of BZW2 also been identified in pancreatic cancer, urothelial cancer, thyroid cancer, and melanoma [11–13]. Gao and colleagues have demonstrated that knockdown of BZW2 inhibits cell growth and regulates cell cycle and apoptosis, indicating BZW2 might be a target for the treatment of muscle-invasive bladder cancer [12]. Another study performed by Cheng and colleagues also suggested that suppression of BZW2 had inhibitory effects on osteosarcoma growth in part by the regulation of the Akt/mTOR signaling pathway [13]. More recently, overexpression of BZW2 has been identified to be associated with hepatoma cell drug resistance, and inhibition of BZW2 inhibits cell proliferation, migration, and invasion by regulating PI3K/Akt/mTOR signaling pathway [11]. All these results suggested BZW2 as a potential target for some types of cancer.

Long-chain non-coding RNA (lncRNA) is non-coding transcript longer than 200 nucleotides. LncRNA is dysfunctional in a variety of human diseases including cancerous to non-cancerous diseases [14, 15]. It plays important roles in many biological and pathological processes by (1) interfering gene transcription and chromatin remodeling, (2) regulating the transcription of target genes, (3) binding and regulating subcellular localization of proteins, and (4) sponging miRNAs [14, 15]. Previous studies have demonstrated that LINC00174 regulated cell proliferation, apoptosis, migration by sponging different miRNAs including miR-152 and miR-138 in glioma therapy [16, 17]. It is interesting to explore the roles of LINC00174 in LP.

To our knowledge, the roles of BZW2 in the LP are still unknown. Therefore, in the present study, for the first time, we investigated the roles of BZW2 in the LP and its underlying molecular mechanisms. Interestingly, we observed that the levels of BZW2 were up-regulated in the LP tissues as compared with the adjacent tissues. The knockdown of BZW2 significantly inhibited cell proliferation and promoted cell apoptosis in the LP cells. Moreover, this study also clarified the underlying mechanisms of BZW2 on the LP.

## Materials and methods

### Clinical specimens and cells

LP and adjacent normal laryngeal (NL) tissue specimens were obtained from patients with LP that enrolled in the Second Xiangya Hospital between 2017 and 2018. The protocol used in the present study has been approved by the Second Xiangya Hospital ethical committee and all patients have read and signed informed consent. LP and NL cells were isolated from the LP and NL tissues, respectively. After that, the cells were cultured in the complete medium containing 100 μg/ml streptomycin, 100 IU/ml penicillin, and 10% fetal bovine serum (Gibco, Grand Island, NY), at 37 °C in the presence of 5% CO_2_.

### Construction of knocked down, mutated, and overexpressed cell lines

The cells were seeded into the indicated plate and when the cell confluency reaches 60–70%, siRNAs were transfected into the cells using the Lipofectamine reagents according to the instructions of the manufacturers. The sequences for siRNAs are shown as follows: BZW2 siRNA1: 5′-GGA GGA ACG CGC ATA GAT GAT-3, BZW2 siRNA2: 5′-GAA ACA GCA GTG ATT GGT CTT-3′, LINC00174 siRNA1: 5′-GAT GTC TCA CCC TAC TCT CTA-3′, and LINC00174 siRNA2: 5′-GCT GAC TGC GAA ACG AAG TGA-3'.

To construct the overexpressed LINC000174 cell lines, the primers were used to amplify LINC000174 gene shown as follow: LINC00174 forward: 5′-CTA GAA TTC GTG GTT TGA TCT TGG CTC ACT GCA-3′; and reverse: 5′-CTA GGA TCC TTT AAA ACA GAA ATC ACT GAA ACC AC-3′. Then the sequences were sub-cloned into a PMX-3 × Flag empty vector. The cells were then transfected with the constructed vectors After the selection of using antibiotics, the stable cell lines were acquired.

To construct mutated cells, the primers used for amplification of BZW2 3′UTR and LINC00174 are shown as follows: BZW2 3′UTR forward: 5′-ATT ATA CTT GGG ACT CGA GCG GT-3, and reverse: 5′-ACC GCT CGA GTC CCA AGT ATA AT. LINC00174 forward: 5′-CCG CCC CAC CGG ACG ACC CCC-3′, and reverse: 5-GGG GGT CGT CCG GTG GGG CGG-3′. After that, the PCR product was inserted into a vector and then transfected into the cells.

### Oligonucleotides transfection

Oligonucleotides transfection was performed according to previously reported methods. In brief, the inhibitor negative control (NC-inhibitor), miR-4500 inhibitor, miR-4500 mimics, mimic negative control (miR-NC) were obtained from GenePharma (Shanghai, China) and transfected into cells using Lipofectamine 2000 (Invitrogen).

### CCK-8 and colony formation assays

To evaluate the cell viabilities and proliferation, cell-counting kit 8 (CCK-8) and colony formation assays were used. In brief, after the cells were transfected with the indicated siRNAs, they were incubated with CCK-8 solution for 1 h. After that, the plates were read at 450 nm using a microplate reader. For colony formation assay, after treatment of siRNA, the cells were counted and seeded in the plates. Next, the cells, fixed using methanol, were stained with crystal violet solution. After washing with phosphate-buffered saline, the cells were observed under light microscopy and cell numbers were counted.

### Quantitative caspase-3 activity and nucleosomal fragmentation assays

Quantitative caspase-3 activity and nucleosomal fragmentation assays were used to evaluate cell apoptosis. Nucleosomal fragmentation assay was performed according to the manufactures' instructions. The absorbance of treatment groups was normalized to that of an un-treated group. For quantitative caspase-3 activity assay, after the cells were treated, lysis buffer was added and then the supernatant was collected. After the substrate was added, cleavage reagent was added to detect the release of pNA by using a microplate reader at a wavelength of 405 nm.

### Quantitative real-time reverse transcription (qRT)-PCR

RNA extraction kit was used to isolate RNA from the cells, according to the manufacturer's document. Reverse transcriptase was used in the RT reaction. The primers for the target genes are shown as follows. BZW 2 forward: 5′-TTT CTG GAC TCT ACA GGC TCA A-3′, and reverse: 5′-ACC ATC ATC TAT GCG CGT TCC-3′; LINC00174 forward: 5′-GGC CCA ACA CTT CCC TCA AA-3′ and reverse: 5′-CAG GGA GAA ACG ACC TGG AG-3′; miR-4500 forward: 5′-TGA GGT AGT AGT TTC TTG CGC C-3′, and reverse: 5′-GTG CAG GGT CCG AGG T-3′; GAPDH forward: 5′-TGA CTT CAA CAG CGA CAC CCA-3′, and reverse: 5′-CAC CCT GTT GCT GTA GCC AAA-3′. The Melt curves were used in order to analyze the accuracy. The expressions of each gene were calculated using 2^−△△Ct^ values. The mRNA expression values of target genes were normalized to that of U6 or GAPDH.

### Western blot

The protein was extracted according to the previous methods [18]. In brief, a cold RIPA buffer containing protease inhibitor was used to lyse the cells or tissue. After that, the extraction buffer was centrifuged (12,000*g*, 10 min) to remove the cell debris and other insoluble materials. The BCA protein assay kits were applied to qualify the concentrations of extracted proteins.

An equal amount of proteins was loaded and separated using the 10% SDS gel. After that, the gel was transfer to a PVDF membrane, which was blocked with 5% non-fat milk at RT for 2 h. Next, a primary antibody against anti‐BZW2 (Sigma, St. Louis, MO) (1:300), or anti‐ACTIN (Santa Cruz Biotechnology Inc, Santa Cruz, CA) (1:2000) was used to incubate with the membrane at 4 °C overnight. Appropriated secondary antibodies conjugated with HRP were used and the imaging system was applied to qualify the expressions of each target proteins.

### Luciferase assays

Luciferase assays were applied to investigate the interactions between BZW 3′ UTR and miR4500, and interactions between LINC00714 and miR4500, in the cells. In brief, when the cells reach 60–70% confluency, the cells were co-transfected with the luciferase reporter plasmids containing wild-type or mutant plasmids and miR-4500 or miR-negative control (NC). The activities of luciferase were determined after the transfection of 24 h.

### Biotin-coupled miRNA pulldown assay

Biotin-coupled miRNA pulldown assay was performed according to a previously reported method [19]. In brief, biotin-coupled miR-4500 was used to incubate with the cell lysates of LP cells. After that, the RNA-RNA complexes were isolated using streptavidin-coupled agarose beads. All experimental procedures were performed under a RNase free condition.

### Statistical analysis

Data were shown as mean ± S.D. Student’s t test, one- or two-way analysis of variance (ANOVA) with multiple comparisons and Student–Newman–Keuls (SNK) test were performed. A *P*-value that less than 0.05 was thought as a statistical significance between two groups. Biochemical studies were repeated at least 3 times.

## Results

### The levels of BZW2 were up-regulated in the laryngeal papilloma tissues

**Fig. 1:**
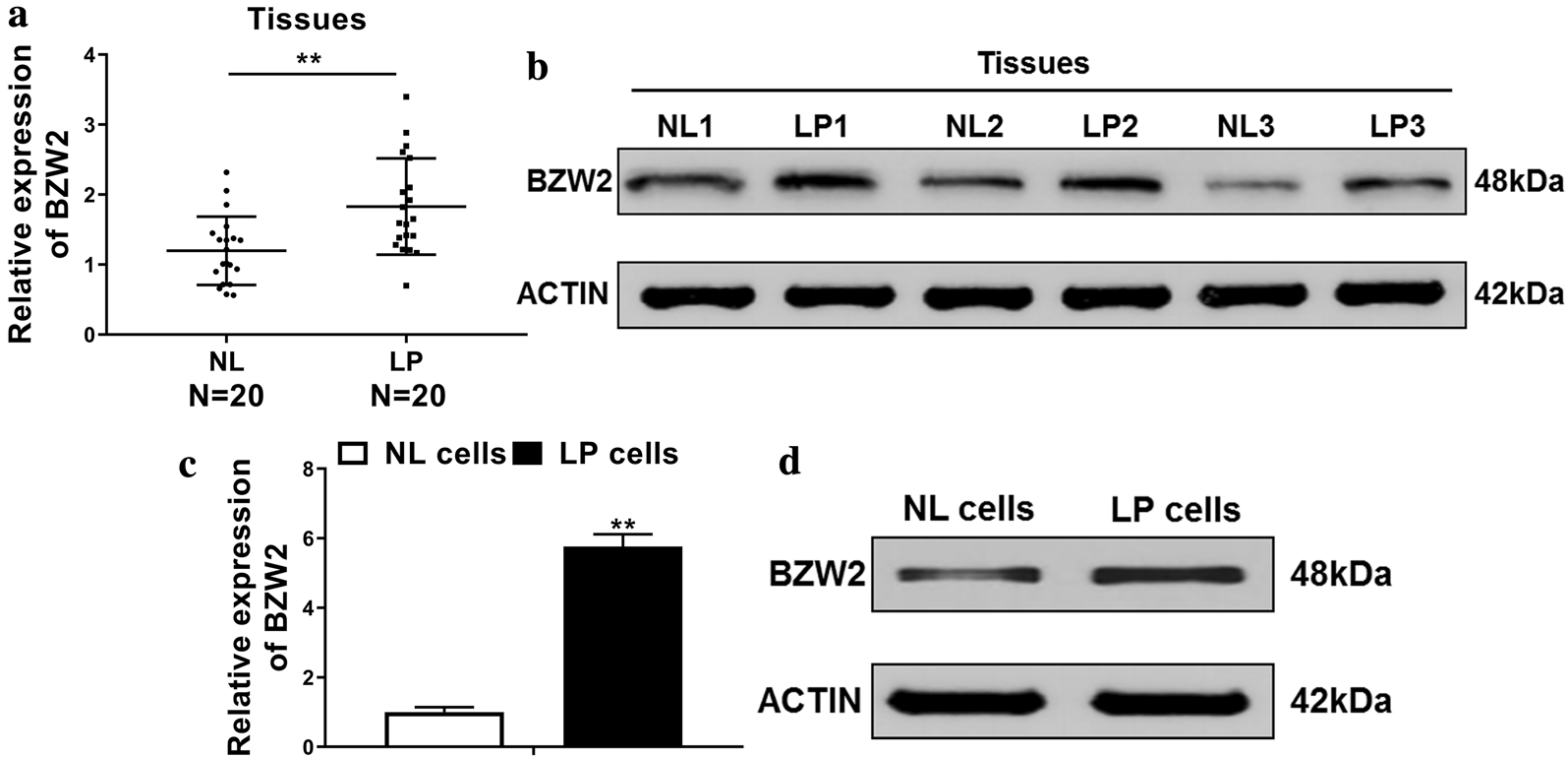
The levels of BZW2 were up-regulated in the laryngeal papilloma tissues. **a**, **b** qRT-PCR and Western blot were employed to analyze the mRNA and protein levels of BZW2 in laryngeal papillomatosis (LP) and adjacent normal laryngeal (NL) tissues. In particular, the protein expressions of BZW2 are from three representative LP and NL. **c**, **d** Private cells were isolated from LP and NL, respectively, and the mRNA and protein expressions were determined using qRT-PCR and western blotting, respectively. *P < 0.05, **P < 0.01

We first investigated the levels of BZW2 in LP and NL tissues. The results showed that the relative expressions of BZW2 in the LP tissues were up-regulated as compared with those in the NL tissues (Fig. 1a). Besides, western blot demonstrated that the protein expressions of BZW2 in the LP tissues were also higher than those in the NL tissues (Fig. 1b). In addition, we observed that the mRNA and protein levels of BZW2 were obviously increased in the LP cells as compared with those in the NL cells (Fig. 1c, d).

### Knockdown of BZW2 promoted cell apoptosis and inhibited cell proliferation

**Fig. 2:**
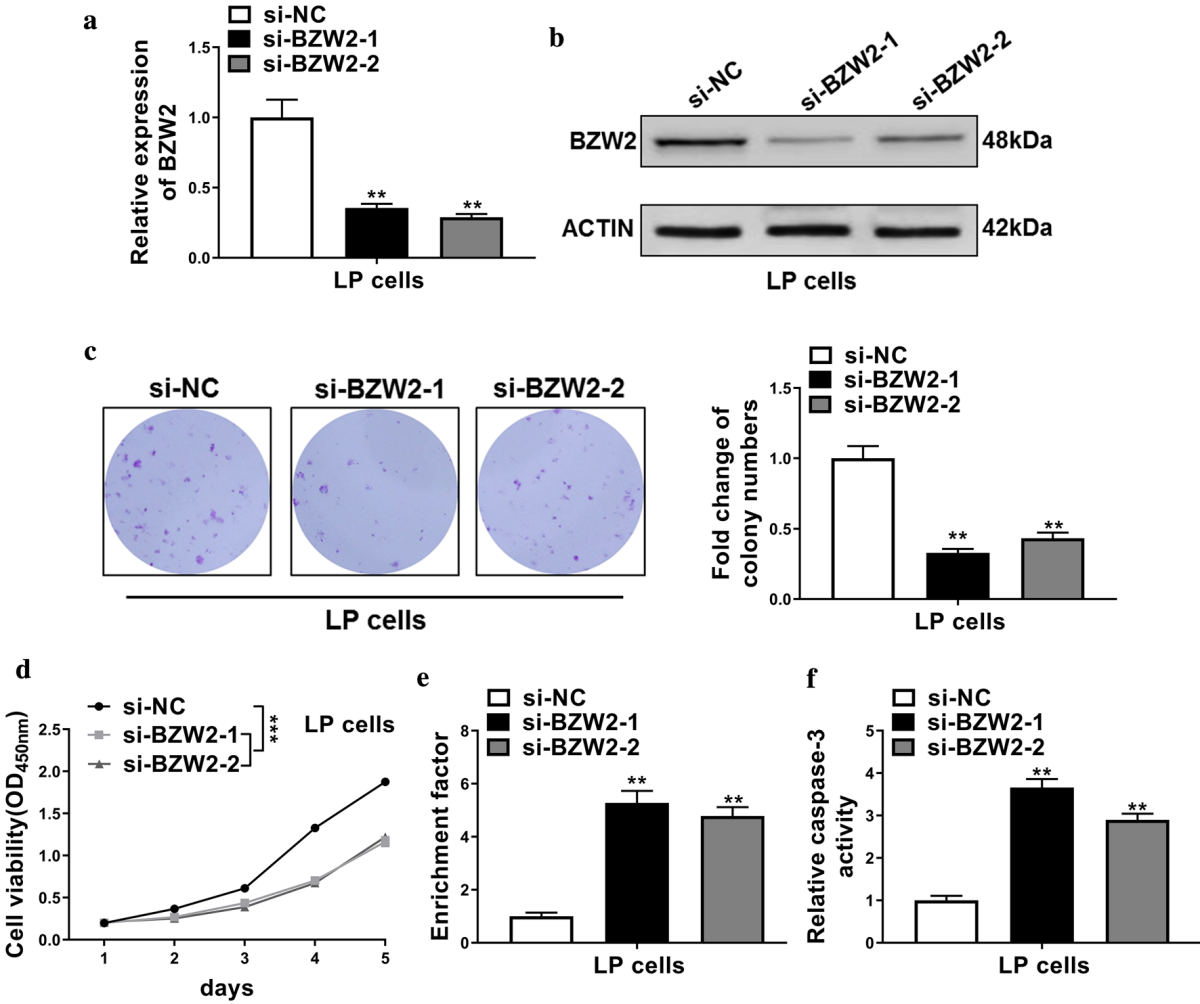
Knockdown of BZW2 inhibited cell proliferation and promoted cell apoptosis. **a**, **b** qRT-PCR and Western blot were employed to analyze the mRNA and protein levels of BZW2 in LP cells that were transfected with negative control siRNA (si-NC), or BZW2 siRNAs including si-BZW2-1 and si-BZW2-2. **c**, **d** Colony formation and CCK-8 assays were used to evaluate cell proliferation in each group. **e**, **f** Nucleosomal fragmentation and caspase-3 activity assays were used to determine cell apoptosis in each group. The data represented are the means ± S.D. *P < 0.05, **P < 0.01, *** *P* < 0.001

We knocked down the BZW2 using BZW2 siRNAs including si-BZW2-1 and si-BZW2-2. The mRNA and protein levels of BZW2 were obviously decreased in those cells transfected with BZW2 siRNAs (Fig. 2a, b). Next, the effects of BZW2 knockdown on cell proliferation and apoptosis were further investigated. The results showed that the numbers of colonies in LP cells that were transfected with BZW2 siRNAs were significantly decreased (Fig. 2c). Similarly, cell viabilities of LP cells that were transfected with BZW2 siRNAs were also decreased (Fig. 2d). These results demonstrated that knockdown of BZW2 inhibited cell proliferation.

The nucleosomal fragmentation assay demonstrated that the enrichment factor of LP cells that were transfected with BZW2 siRNAs were increased (Fig. 2e). Additionally, we noticed the LP cells that were transfected with BZW2 siRNAs with higher caspase-3 activities as compared with those in cells that were transfected with si-NC (Fig. 2f). These results demonstrated that knockdown of BZW2 promoted cell apoptosis.

### The expressions of BZW2 were negatively regulated by miR-4500

**Fig. 3:**
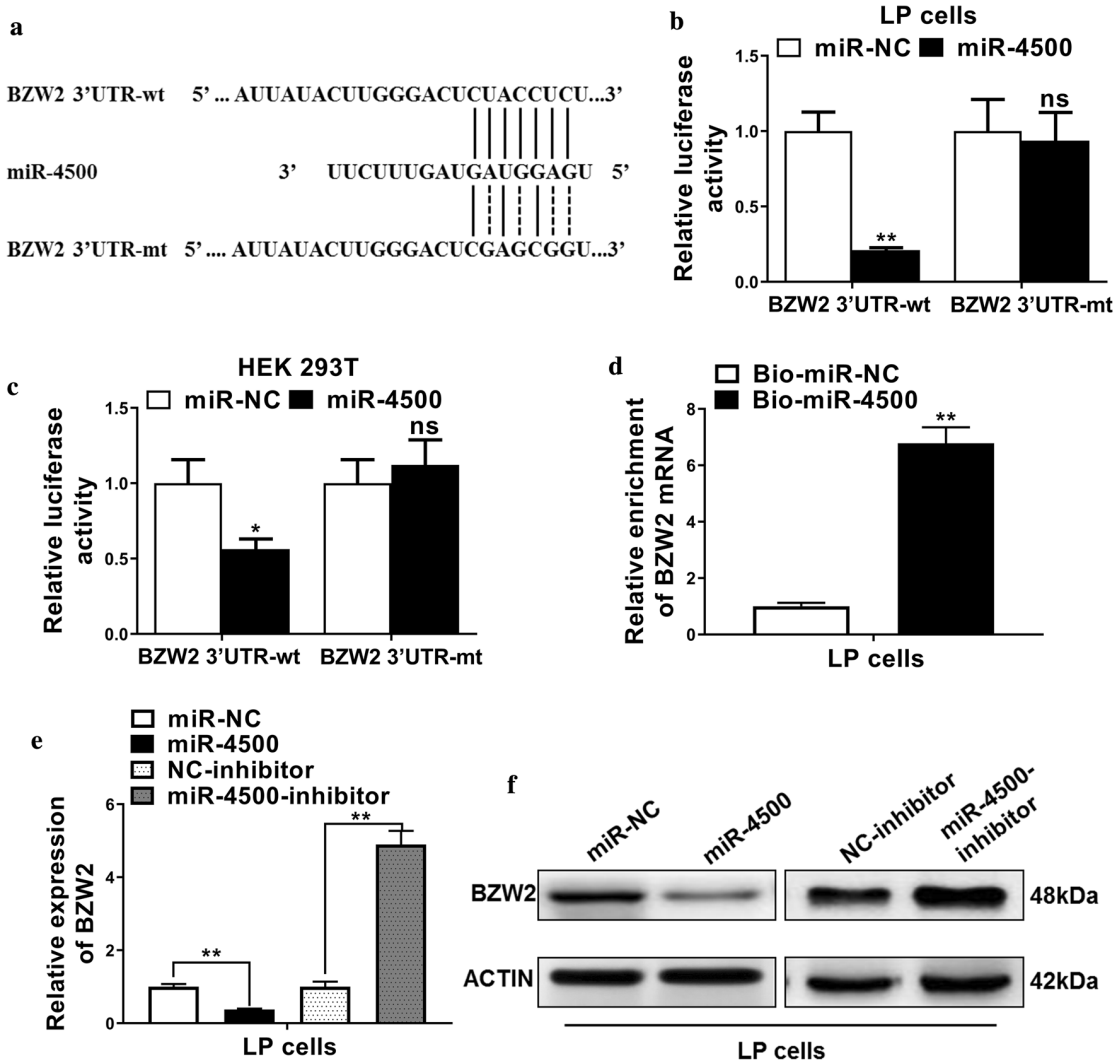
The expressions of BZW2 were negatively regulated by miR-4500. **a** Schematic diagram showed predicted binding sites between wild-type or mutated miR-4500 and the 3′UTR of BZW2. **b**, **c** Luciferase reporter was used to determine the relative luciferase activities in cells that were transfected with plasmids containing BZW2 3′UTR-wide type (wt) or mutant (mt) and miR-4500 mimics or miR-NC. **d** Biotin-coupled miRNA pulldown assay was used to determine enrichment of BZW2 mRNAs in the RNA-RNA complexes. **e**, **f** qRT-PCR and western blotting were used to determine the mRNA and protein levels of BZW2 in each group. The data represent the mean ± SD. *P < 0.05, **P < 0.01, ns indicates no significance

We identified that miR-4500 was a target of BZW2 and their predicted binding sites as shown in Fig. 3a. We then used a luciferase reporter assay to verify the interactions between BZW2 and miR-4500 in the LP and HEK293T cells. We found that luciferase activities were significantly decreased in cells that were co-transfected with BZW2 3-UTR-wt plus miR-4500, whereas the luciferase activities in cells that were co-transfected with BZW2 3-UTR-mt plus miR-4500 did not show the significant difference as compared with cells that were BZW2 3-UTR-mt plus miR-NC (Fig. 3b, c). To confirm the interactions between BZW2 and miR-4500. Biotin-coupled miRNA pulldown assay was used to determine the enrichment of BZW2 mRNAs in the RNA-RNA complexes. The results showed enrichment of BZW2 mRNAs in the Bio-miR-4500 complexes (Fig. 3d).

Next, we determined the mRNA and protein levels of BZW2 in each group. We found that the cells transfected with miR-4500 have lower expressions of BZW2, whereas the application of miR-4500 inhibitor boosted the expressions of BZW2 (Fig. 3e, f). These results supported that expressions of BZW2 were negatively regulated by miR-4500.

### LINC00174 competed with the BZW2 for binding with miR-4500

**Fig. 4:**
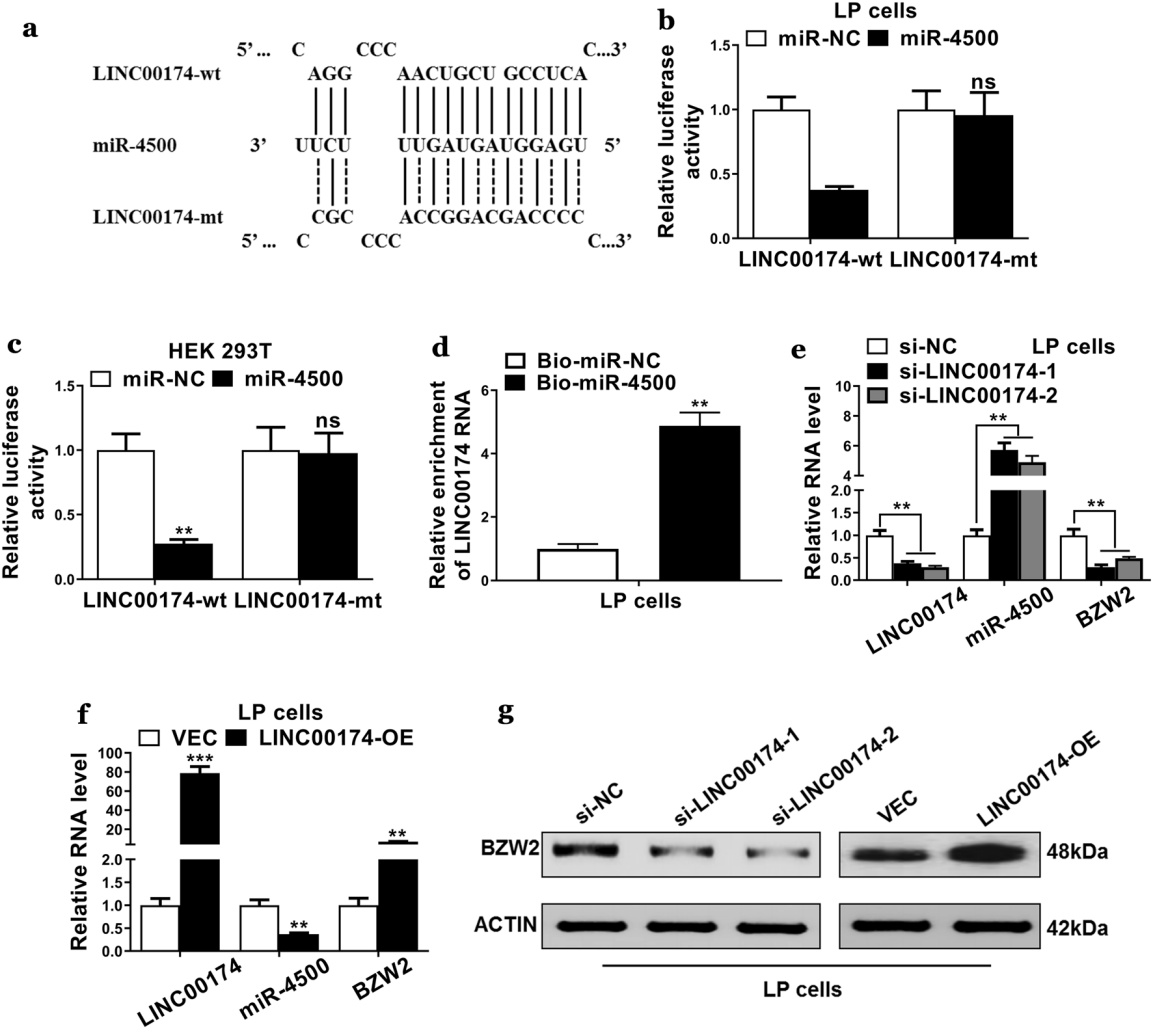
LINC00174 competed with the BZW2 for binding with miR-4500. **a** Schematic diagram showed predicted binding sites between wild-type or mutated LINC00174 and miR-4500. **b**, **c** Luciferase reporter was used to determine the relative luciferase activities in cells that were transfected with plasmids containing LINC00174-wt or mt and miR-4500 or miR-NC. **d** qRT-PCR was used to determine relative enrichment of LINC00174 RNAs in the RNA-RNA complexes. **e**, **f** qRT-PCR was used to determine the mRNA levels of genes including LINC00174, miR-4500, and BZW2 in LP cells that were transfected with LINC00174 siRNAs (si-LINC00174-1 and si-LINC00174-2) or si-NC and in the LP cells that were transfected with LINC00174 overexpression plasmids (LINC00174-OE) or empty plasmid vector (VEC). **g** The protein expressions of BZW2 were determined using western blot in LINC00174 knockdown or LINC00174 overexpressed cells. The data represented are the means ± S.D. *P < 0.05, **P < 0.01, ns indicates no significance

We next sought the target of miR-4500. LINC00174 was identified and predicted binding sites between miR-4500 and LINC00174 were shown in Fig. 4a. We then applied a luciferase reporter assay to verify the interactions between LINC00174 and miR-4500 in the LP and HEK293T cells. We found that luciferase activities were significantly decreased in cells that were co-transfected with LINC00174-wt plus miR-450, whereas the luciferase activities in cells that were co-transfected with LINC00174-mt plus miR-4500 did not show the significant difference as compared with cells that were BZW2 3-UTR-mt plus miR-NC (Fig. 4b, c). Biotin-coupled miRNA pulldown assay showed enrichment of BZW2 mRNAs in the Bio-miR-4500 complexes (Fig. 4d). Next, we knocked down the LINC00174 in the LP cells. Interestingly, the results showed the mRNA levels of miR-4500 were significantly increased (Fig. 4e). However, when we overexpressed the LINC00174 in the LP cells, the mRNA levels of miR-4500 were decreased and the mRNA and protein levels of BZW2 were significantly increased (Fig. 4f, g). These results supported that LINC00174 competed with the BZW2 for binding with miR-4500.

### LINC00174/miR-4500/BZW2 axis regulated cell proliferation and apoptosis

**Fig. 5:**
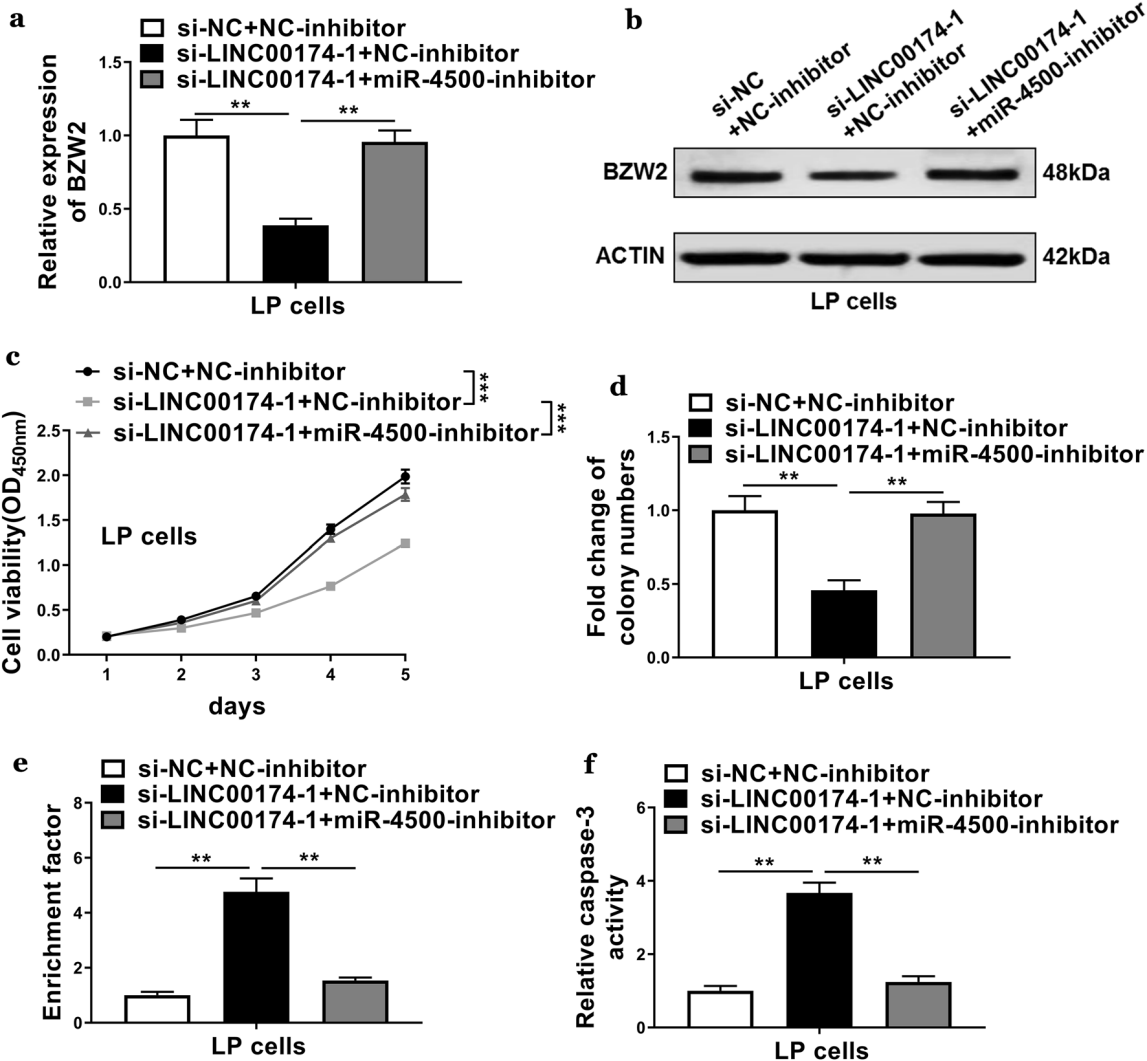
LINC00174/miR-4500/BZW2 axis regulated cell proliferation and apoptosis. The LP cells were co-transfected with si-NC plus miRNA negative control (NC)-inhibitor), LINC00174-1 siRNA plus miRNA NC-inhibitor, or LINC00174-1 siRNA plus miR-4500 inhibitor. **a**, **b** qRT-PCR and western blotting were used to determine the mRNA and protein levels of BZW2. **c**, **d** CCK-8 and colony formation assays were used to evaluate cell proliferation. **e**, **f** Nucleosomal fragmentation and caspase-3 activity assays were used to assess cell apoptosis in each group. The data represented are the means ± S.D. *P < 0.05, **P < 0.01, *** *P* < 0.001

Finally, we evaluated the effects of the LINC00174/miR-4500/BZW2 axis on the regulation of cell proliferation and apoptosis. First, we determined the expressions of BZW2 in the si-LINC00174 transfected cells that were treated with NC-inhibitor or miR-4500 inhibitor. Interestingly, in the LP cells that were co-transfected with si-LINC00174 and miR-4500 inhibitor showed higher mRNA and protein expressions as compared with those in the cells that were co-transfected with si-LINC00174 and NC-inhibitor (Fig. 5a, b), indicating suppression of miR-4500 able to recover the expressions of BZW2. We then determined the cell viabilities and apoptosis in those transfected cells. The results showed that knockdown of LINC00174 decreased the cell viabilities and colony numbers, whereas suppression of miR-4500 recovered the cell viabilities and colony numbers (Fig. 5c, d). Moreover, the enrichment factor and caspase-3 activities were enhanced in the cells transfected with si-LINC00174 plus NC-inhibitor, whereas suppression of miR-4500 decreased the cell apoptosis (Fig. 5e, f). These results demonstrated the abilities of the LINC00174/miR-4500/BZW2 axis to regulate cell proliferation and apoptosis.

## Discussion

Once LP spreads, it is very difficult to remove all tumor tissues, thereby leading to its recurrence. Besides, the recurrence of LP leads to its tenacious drug resistance [20]. It is urgent to explore the underlying mechanisms of the occurrence and development of LP.

It is known that BZW regulates the transcriptions of histone H4 and plays the important roles in cell differentiation [10]. Previous studies have demonstrated that high expressions of BZW2 in a series of cancers including pancreatic cancer, urothelial cancer, thyroid cancer, and melanoma [11, 12]. However, to our knowledge, the expression patterns of BZW2 in the LP are still unknown. Herein, the current investigation, for the first time, explored the expression patterns of BZW2. The results demonstrated that BZW2 served as an oncogene and able to regulate cell proliferation and apoptosis in the LP. BZW2 levels, both at the mRNA and protein levels, were significantly increased in the LP tissues as compared with those in the NL tissues. The knockdown of BZW2 decreased cell proliferation and apoptosis.

miRNAs also could be a target that is regulated by other genes [7, 21]. In this study, miR-4500 was identified as a target of BZW2. Previous studies have demonstrated the roles of miR-4500 in the occurrence and development of various cancers including hepatoma, non-small cell lung cancer (NSCLC) and colorectal cancer [22–25]. For instance, Zhang and colleagues have demonstrated the low expressions of miR-4500 associated with cancer cell proliferation in NSCLC [22]. Suppression of miR-4500 is an effective strategy to promote lung cancer cell proliferation [22]. Another study performed by Yu and colleagues reported that miR-4500 serves as a tumor suppressor in colorectal cancer [23]. In the present study, we predicted the binding sites between miR-4500 and BZW2 and found miR-4500 negatively associated with BZW2 in the LP cells. In the cells transfected with miR-4500, lower expressions of BZW2 were observed, whereas the application of miR-4500 inhibitor boosted the expressions of BZW2.

We further explored the underlying mechanisms of BZW2 in the LP. As mentioned, miR-4500 was negatively associated with BZW2 in the LP. By combining miRcode and RNAhybrid, LINC00174 was identified as a target of miR-4500. In 2018, Do and Kim for the first time reported that abnormal expressions of LINC00174 are associated with the occurrence of colorectal cancer [26]. Another study initiated by Shen and colleagues then demonstrated that LINC00174 promotes colorectal cancer by regulating miR-1910 [27]. More recently, Shi and colleagues have reported that LINC00174 promotes cell proliferation, migration and apoptosis in part by the regulation of miR-152 in glioma [16]. In the present study, we found the expressions of miR-4500 was regulated by LINC00174. The knockdown of LINC00174 increased the levels of miR-4500, whereas overexpression of LINC00174 suppressed the levels of miR-4500. Interestingly, the expressions of BZW2 was also affected by the expressions of LINC00174. When we overexpressed the LINC00174 in the LP cells, BZW2, both at the mRNA and protein levels, were significantly increased, indicating that LINC00174 competed with the BZW2 for binding with miR-4500.

Many recent studies had shed light on the roles of BZW2 in cancers [11–13]. For instance, Gao and colleagues have demonstrated that suppression of BZW2 inhibits cell growth and regulates cell cycle and apoptosis, indicating BZW2 might be a target for the treatment of muscle-invasive bladder cancer [12]. Cheng and colleagues have reported that suppression of BZW2 exists inhibitory effects on osteosarcoma growth in part by the regulation of the Akt/mTOR signaling pathway [13]. More recently, overexpression of BZW2 has been identified to be associated with hepatoma cell drug resistance, and inhibition of BZW2 inhibits cell proliferation, migration, and invasion by regulating PI3K/Akt/mTOR signaling pathway [11]. However, the effects of BZW2 in the occurrence and development of LP and its underlying mechanism are still unknown. The significance of this study is that we firstly identified BZW2 served as an oncogene in LP. Recently, BZW2 has been recognized to be involved in the osteosarcoma by the regulation of Akt/mTOR signaling pathway [13]. Besides, high expression of BZW2 is observed in the urothelial cancer [28]. More recently, Gao and colleagues revealed that the knockdown of BZW2 suppresses cell growth, G1 arrest and apoptosis in the muscle‐invasive bladder cancers [12]. Herein, we reported high expression of BZW2 in LP. Furthermore, we explored the underlying mechanisms of BZW2, which are associated with LINC00174 and miR-4500. We found that the LINC00174/miR-4500/BZW2 axis regulated cell proliferation and apoptosis. These results also highlighted another significance of this study. Previous studies have demonstrated that LINC000174 is associated with miR-138 [17], miR-152 [16], and miR-320 [29]. Herein, we revealed the relationship between LINC00174 and miR-4500. Knockdown of LINC00174 decreased cell proliferation and apoptosis, whereas suppression of miR-4500 recovered those effects. These results supported that targeting the LINC00174/miR-4500/BZW2 axis is an effective strategy to suppress LP cell proliferation and regulate cell apoptosis. Taken together, our study identified the important roles of the LINC00174/miR-4500/BZW2 axis in the LP and might be served as potential targets for the treatment of LP.

## Conclusion

The present study demonstrated that the levels of BZW2 were up-regulated in the LP tissues and cells. The expressions of BZW2 were negatively regulated by miR-4500 and LINC00174 competed with the BZW2 for binding with miR-4500. These results showed that LINC00174/miR-4500/BZW2 axis regulated cell proliferation and apoptosis in the LP, indicating that suppression of BZW2 might be used as a strategy for the treatment of LP caused by the HPV infection.

## Data Availability

They could be achieved upon reasonable request to the authors.

## Abbreviations

BZW: Basic leucine zipper and W2 domains
LP: Laryngeal papilloma
HPV: Human papillomavirus
miRNAs: MicroRNAs
CCK-8: Cell-counting kit 8
NC: Negative control
ANOVA: Analysis of variance
NSCLC: Non-small cell lung cancer
